# Derivation of DO-BITS, a score for predicting success on *Love Island*: a retrospective, observational cohort study

**DOI:** 10.2144/fsoa-2020-0168

**Published:** 2020-11-13

**Authors:** Alex Simpson, Sophie Graham, Cormac Sammon, Thomas P Leahy, Sreeram V Ramagopalan

**Affiliations:** 1 Bristol Myers Squibb, Uxbridge, UK; 2 Evidera, London, UK; 3 PHMR, London, UK; 4 F Hoffman-La Roche, Basel, Switzerland

**Keywords:** DO-BITS, *Love Island*, prediction, television, winning

## Abstract

**Objective:**

To derive a score for finishing in the top three positions of the television show, *Love Island*, UK.

**Design:**

A retrospective study was undertaken using data from all previous contestants.

**Results:**

A score predicting show success termed DO-BITS (different coupling approaches [islanders pursuing one or many people on the show], Original islander [being on the show from the start], [being] Brunette, [having] intimate relationships on screen, Tradesman [occupation before being on the show] and Short name [having a four-letter first name]) was developed. The accuracy of this score in this derivation cohort yielded a C-statistic of 0.85.

**Conclusion:**

This simple, novel score provides a practical tool to assess the likelihood of success on *Love Island*.

## Lay abstract

*Love Island* is a reality television program that has attracted a lot of attention, including participation from medical and scientific professionals. In order to help people decide whether to potentially make a life changing move to participate on the show, we sought to identify predictors of show success. Our results enabled the generation of a simple score called DO-BITS that provides a practical tool to assess the individual likelihood of success on *Love Island*, potentially aiding decision making regarding whether or not to apply to participate on the show and/or behavior as a contestant on the show.

*Love Island* is a reality television program that was originally aired by ITV2 in the UK in 2015 [1]. The premise of the show is that contestants (‘islanders’) live in isolation in a villa and are constantly being recorded. To continue participating in (‘surviving’) the show, islanders have to form relationships (‘couple up’) with other contestants and be voted to stay in either by fellow islanders or members of the public. Relationships are ideally for love but can be for friendship. Developing relationships (‘cracking on’ or ‘grafting’) is the key tactic to achieving success in the show, and islanders need to avoid relationships ending (getting ‘pied’ or ‘mugged off’). At the start of the show, couples are formed based on first impressions, but these couples may change over time (‘recoupling’), if preferences change (‘heads turned’). Over the course of the show, couples are gradually eliminated (although new islanders may enter, and occasionally eliminated islanders can re-enter) and at the final, the couple with the most votes is awarded a cash prize of £50,000. Further, the length of time spent on the show is linked to postshow earnings, which have been estimated to have a lifetime added value greater than obtaining a degree from Oxford or Cambridge Universities [2]. As such, the show has attracted the participation of medical and scientific professionals. Since the show was first aired, there have been six series, with the villa being in Mallorca for series 1–5 (broadcast during UK summer) and in South Africa for series six (broadcast during UK winter). Data on the winners and runners up for each of the series is publicly available, as well as data on the characteristics of each contestant. Using this data, we sought to derive a score for show success (i.e., finishing in the top three) based on contestant characteristics, as it could assist healthcare professionals and researchers who are deciding whether or not to apply to participate in *Love Island* 2021.

## Materials & methods

### Study design & population

We undertook a retrospective cohort study using data from all islanders participating in the six series of *Love Island*, UK, from 2015 to 2020.

### Ascertainment of outcomes & characteristics

Our primary outcome measure was finishing in the top three positions of the show for each series which was obtained from the *Love Island* Fandom Wiki [3]. As the top three positions are only awarded to couples, there were six islanders classed as having the outcome in every series.

The authors were unable to identify peer-reviewed literature to guide predictor inclusion and therefore two approaches were taken to identify potential predictors. First, the *Love Island* Fandom Wiki was used to obtain data on readily available potential predictors including name, age at time of villa entry, sex, home town, day of entry to the villa (in relation to series start), occupation prior to show entry and number of couplings during the islander’s time on the show. Second, predictors deemed relevant by the authors were identified through Google searches. Data to determine whether an islander had intimate relationships on screen (‘did bits’) came from a newspaper [4]. Data regarding ethnicity was obtained from reviewing footage and images of the islanders as well as Google searches. Ethnicity was classified as White, Black or Asian. Where an islander was mixed race, they were assigned to the minority race. Smoking on screen (for series 1–3 only as for series 4–6 smokers had to smoke outside of the villa in isolation), was assessed by reviewing *Love Island* episodes. Islander star sign (where available) was obtained from the Famous Birthdays webpage [5].

Using islander name, we derived potential predictors of first name being four letters, first name being five letters and first letter of first name being in the first half of the alphabet. Using home town we derived potential predictors of living in London, the County of Essex or the South of England (as defined here [6]. Using occupation we derived potential predictors of being a healthcare professional (nurse, physiotherapist, doctor or pharmacist), being a public servant (nurse, physiotherapist, doctor, pharmacist, civil servant, fire fighter or police officer), being a tradesman (builder, joiner, scaffolder, carpenter, mechanic, heating engineer, plumber, construction worker or glazier), and being a model or having a sports related occupation (personal trainer, professional sports player). Using day of entry into the villa we derived a predictor of being an original islander (being on the show from the start of the series).

We included variables that were related to activity after entry on the show – for example, doing bits and number of couplings as these have the potential to guide island behavior if they decide to participate in the show. We analyzed these variables in terms of the potential strategy for the contestant (e.g., doing bits or not). For relationships, as there are two potential strategies–pursuing one person (‘eggs in one basket’) or grafting many people. The category for the association analysis was therefore based on testing the effect of having a strategy versus not.

### Statistical analysis

The sample population consistent of 187 contestants, of which 36 had the primary outcome of interest. Continuous variables were categorized after inspection of the data. Descriptive statistics were used to describe characteristics of the overall population and the population stratified by the primary outcome. A univariate, complete case, logistic regression analysis was conducted to assess which predictors were associated with the outcome. As no published literature exists on predictors for success, we used a statistical significance threshold for variable inclusion in to multivariable analyses. All potential predictors identified from the univariate analyses with a p-value < 0.1 were used in a multivariable logistic regression analysis, to create a score for success on the show. The α = 0.1 level was chosen due to the limited size of the dataset and therefore by increasing the threshold, it will increase the chance of detecting differences. Odds ratios and 90% CI were calculated for all univariate and multivariate associations with the primary outcome. Performance of the resulting score was assessed by the relative operating characteristic and the calculation of the C-statistic.

All analyses were conducted in R version 3.5.3.

## Results

### Population characteristics

Over all six series of the show, there were 187 islanders, with 36 islanders finishing in the top three positions. Characteristics of all participants and those finishing in the top three positions are shown in Table 1.

**Table 1.** Islander characteristics, overall and stratified by finishing in the top three positions.

Predictor		All contestants, n = 187	Contestants that did not make top three, n = 151	Top three contestants, n = 36
		n (%)	n (%)	n (%)
Age category (years)	17–22	72 (39%)	57 (38%)	15 (41%)
	22–26	82 (44%)	68 (45%)	14 (38%)
	26–31	33 (18%)	26 (17%)	7 (19%)
Hair color on screen	Black	47 (25%)	41 (27%)	6 (17%)
	Blonde	55 (29%)	47 (31%)	8 (22%)
	Brown	84 (45%)	63 (42%)	21 (58%)
	Red	1 (1%)	0 (0%)	1 (3%)
Ethnicity	White	149 (80%)	118 (78%)	31 (86%)
	Asian	5 (3%)	4 (3%)	1 (3%)
	Black	33 (18%)	29 (19%)	4 (11%)
Sex	Female	92 (49%)	74 (49%)	18 (50%)
	Male	95 (51%)	77 (51%)	18 (50%)
Number of couplings	0	36 (19%)	36 (24%)	0 (0%)
	1	75 (40%)	59 (39%)	16 (44%)
	2	30 (16%)	22 (15%)	8 (22%)
	3	21 (11%)	19 (13%)	2 (6%)
	4	18 (10%)	11 (7%)	7 (19%)
	5	6 (3%)	3 (2%)	3 (8%)
	6	1 (1%)	1 (1%)	0 (0%)
Sport related occupation	No	161 (86%)	130 (86%)	31 (86%)
	Yes	26 (14%)	21 (14%)	5 (14%)
Model occupation	No	150 (80%)	122 (81%)	28 (78%)
	Yes	37 (20%)	29 (19%)	8 (22%)
Healthcare occupation	No	180 (96%)	144 (95%)	36 (100%)
	Yes	7 (4%)	7 (5%)	0 (0%)
Public service occupation	No	176 (94%)	140 (93%)	36 (100%)
	Yes	11 (6%)	11 (7%)	0 (0%)
Tradesman occupation	No	171 (91%)	142 (94%)	29 (81%)
	Yes	16 (9%)	9 (6%)	7 (19%)
Home town is outside of England	No	161 (86%)	131 (87%)	30 (83%)
	Yes	26 (14%)	20 (13%)	6 (17%)
Home town is in London	No	153 (82%)	122 (81%)	31 (86%)
	Yes	34 (18%)	29 (19%)	5 (14%)
Home town is in Essex	No	168 (90%)	137 (91%)	31 (86%)
	Yes	19 (10%)	14 (9%)	5 (14%)
Home town is in the South of England	No	82 (44%)	69 (46%)	13 (36%)
	Yes	105 (56%)	82 (54%)	23 (64%)
Five-letter first name	No	141 (75%)	115 (76%)	26 (72%)
	Yes	46 (25%)	36 (24%)	10 (28%)
Four-letter first name	No	143 (76%)	121 (80%)	22 (61%)
	Yes	44 (24%)	30 (20%)	14 (39%)
First letter of first name in first half of alphabet	No	55 (29%)	46 (30%)	9 (25%)
	Yes	132 (71%)	105 (70%)	27 (75%)
Smokes on screen	No	69 (85%)	53 (84%)	16 (89%)
*(Series 1–3 only n = 81)*	Yes	12 (15%)	10 (16%)	2 (11%)
Original islander	No	119 (64%)	103 (68%)	16 (44%)
	Yes	68 (36%)	48 (32%)	20 (56%)
Did bits on show	No	146 (78%)	131 (87%)	15 (42%)
	Yes	41 (22%)	20 (13%)	21 (58%)
Star sign	Aries	7 (4%)	6 (4%)	1 (3%)
	Taurus	21 (11%)	14 (9%)	7 (19%)
	Gemini	12 (6%)	9 (6%)	3 (8%)
	Cancer	20 (11%)	14 (9%)	6 (17%)
	Leo	14 (7%)	10 (7%)	4 (11%)
	Virgo	13 (7%)	11 (7%)	2 (6%)
	Libra	9 (5%)	8 (5%)	1 (3%)
	Scorpio	16 (9%)	14 (9%)	2 (6%)
	Sagittarius	17 (9%)	14 (9%)	3 (8%)
	Capricorn	15 (8%)	12 (8%)	3 (8%)
	Aquarius	13 (7%)	10 (7%)	3 (8%)
	Pisces	16 (9%)	15 (10%)	1 (3%)
	Missing	14 (7%)	14 (9%)	0 (0%)

**Figure 1.**
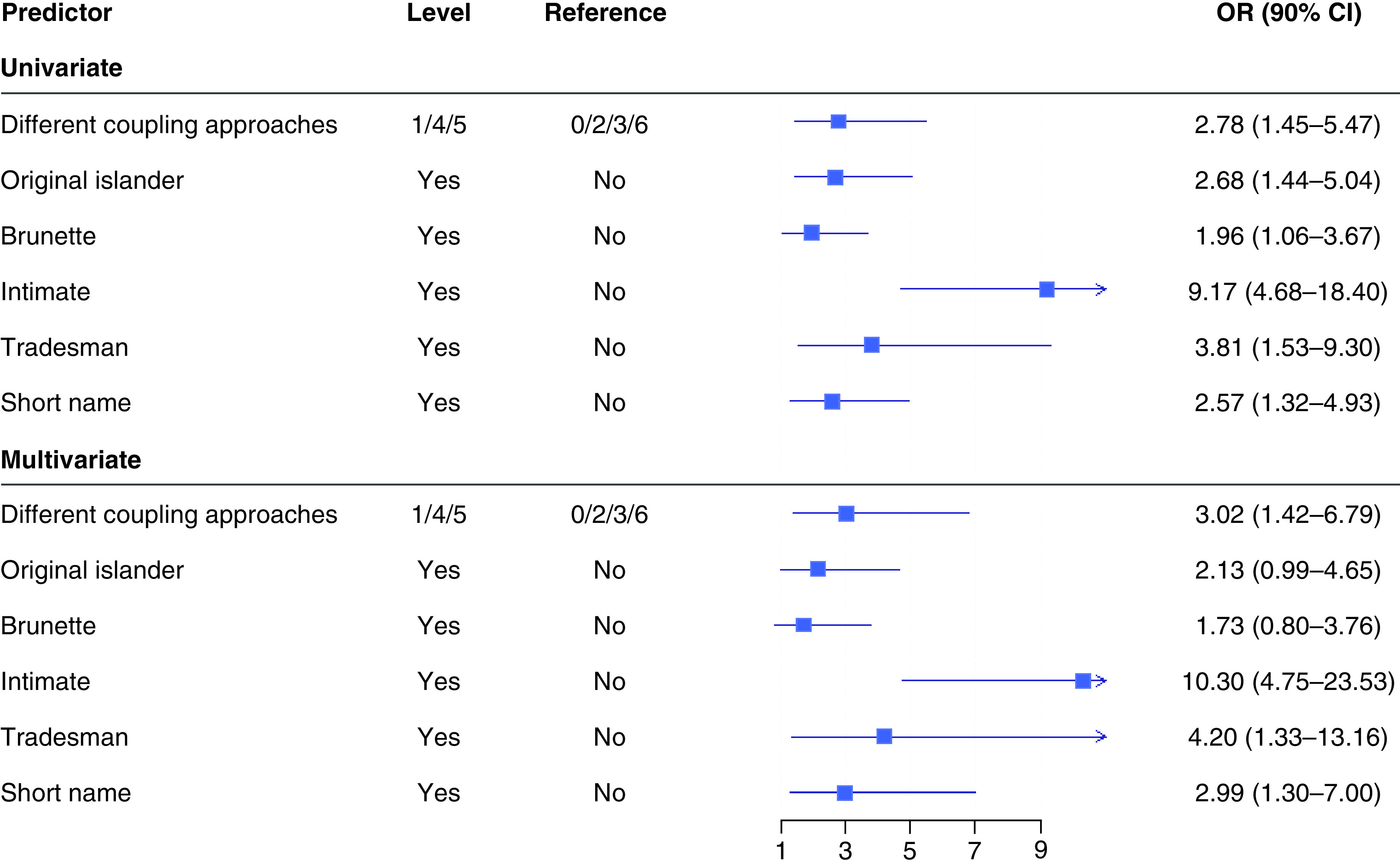
Univariate and multivariable associations of selected characteristics meeting significance threshold with show success. OR: Odds ratio.

Univariate analyses on the association between characteristics and show success is shown in Table 2. Associations with p-values below the defined threshold were observed with brown hair color, having a four-letter first name, being a tradesman, being an original islander, doing bits on screen and coupling strategy. These were taken forward into a multivariable model (Figure 1), where a qualitative assessment of the similarity of associations between the selected predictors from the univariate model was made with the multivariate model. Given the similar magnitudes of association in the multivariate model, all six predictors initially identified through the univariate analyses were used in the DO-BITS score (Table 3).

**Table 2.** Univariate association analysis of potential predictors with show success.

Predictor	Category	Reference	OR (90% CI)	p-value
Age category (years)	17–22	22–26	1.28 (0.64–2.53)	0.55
	26–31	22–26	1.31 (0.54–3.01)	0.6
Hair color on screen	Brown	Not brown	1.96 (1.06–3.67)	0.08
	Blonde	Not blonde	0.63 (0.30–1.26)	0.3
	Black	Not black	0.54 (0.23–1.14)	0.2
	Red	Not red	N/A	0.99
Ethnicity	Asian	White	0.95 (0.09–5.00)	0.97
	Black	White	0.53 (0.19–1.25)	0.26
Sex	Male	Female	0.96 (0.52–1.77)	0.92
Different coupling approaches	1, 4 or 5	0, 2, 3 or 6	2.78 (1.45–5.47)	0.01
Sport related occupation	Yes	No	1.00 (0.38–2.30)	1
Model occupation	Yes	No	1.20 (0.55–2.46)	0.68
Healthcare occupation	Yes	No	N/A	1
Public service occupation	Yes	No	N/A	1
Tradesman occupation	Yes	No	3.81 (1.53–9.30)	0.01
Home town is outside of England	Yes	No	1.31 (0.54–2.92)	0.6
Home town is in London	Yes	No	0.68 (0.27–1.53)	0.46
Home town is in Essex	Yes	No	1.58 (0.59–3.80)	0.41
Home town is in the South of England	Yes	No	1.49 (0.80–2.84)	0.3
Five-letter first name	Yes	No	1.23 (0.60–2.41)	0.62
Four-letter first name	Yes	No	2.57 (1.32–4.93)	0.02
First letter of first name in first half of alphabet	Yes	No	1.31 (0.67–2.72)	0.52
Smokes on screen *(Series 1–3, only n = 81)*	Yes	No	0.66 (0.14–2.29)	0.62
Original islander	Yes	No	2.68 (1.44–5.04)	0.01
Did bits on show	Yes	No	9.17 (4.68–18.40)	<0.01
Star sign	Aries	Cancer	0.39 (0.03–2.23)	0.43
	Taurus	Cancer	1.17 (0.38–3.59)	0.82
	Gemini	Cancer	0.78 (0.18–2.94)	0.76
	Leo	Cancer	0.93 (0.25–3.27)	0.93
	Virgo	Cancer	0.42 (0.08–1.75)	0.35
	Libra	Cancer	0.29 (0.03–1.60)	0.29
	Scorpio	Cancer	0.33 (0.06–1.35)	0.22
	Sagittarius	Cancer	0.50 (0.12–1.80)	0.39
	Capricorn	Cancer	0.58 (0.14–2.13)	0.51
	Aquarius	Cancer	0.70 (0.17–2.61)	0.66
	Pisces	Cancer	0.16 (0.01–0.81)	0.1

**Table 3.** The DO-BITS score.

Variable	Score
(D) Different coupling strategy	1 point if the contestant has been in only one coupling (eggs in one basket) 2 points if the contestant has been in four or five couplings
(O) Original islander	1 point if the contestant enters the villa at series start
(B) Brunette	1 point if the contestant has brown hair on screen
(I) Intimate relationships	1 point if contestant does bits on the show
(T) Tradesman	1 point if the contestant is a tradesman
(S) Short name	1 point if the contestant has a short four-letter first name

**Figure 2.**
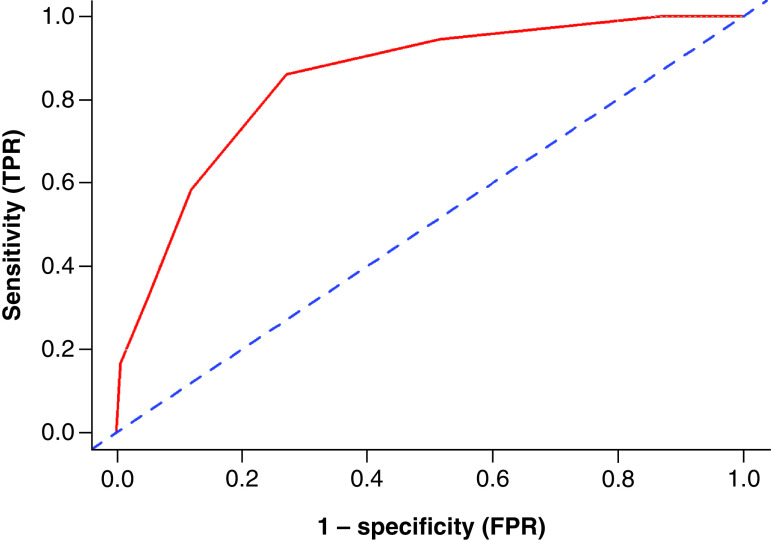
Accuracy of the DO-BITS score in the overall cohort. FPR: False-positive rate; TPR: True-positive rate.

The in-sample accuracy of DO-BITS in the overall population led to a C statistic of 0.85 (Figure 2).

## Discussion

Using a derivation cohort based on the entire population of contestants of *Love Island*, we identified six factors predicting show success (different coupling strategies, original islander, brown hair, intimate relationships, tradesman occupation and short four-letter first name). Using these factors, we developed a novel, user-friendly success score, DO-BITS, which demonstrated good in-sample accuracy in the overall cohort (C statistic 0.85). The score encompasses variables that are somewhat predetermined before applying to be on the show (name, occupation, hair color), although they nevertheless could be changed if an individual wishes to do so before applying. Notably, of the seven healthcare workers that have participated, none have made to the top three suggesting a move into the trades may be advisable for many readers aspiring to become a successful islander. We are unclear as to the process that *Love Island* uses to decide who will be on the show on the first day of any series, but assuming this is out of contestant’s hands, there are two variables that are controllable to a certain extent by participants (whether to do bits on screen or to have coupling strategy that is all eggs in one basket (one coupling) or grafting (four or five couplings). We believe the DO-BITS score will therefore be useful for individuals considering applying to be on *Love Island*, and for islanders on the show who may be thinking of a strategy for success on the show.

### Strengths & limitations

The utility of any tool for risk prediction depends on the quality of the data used to derive it. We only used data that was relatively easy to find online and/or that we specifically searched for. There may be other predictors of success that exist, that we did not investigate because they were harder to discover or unavailable online. We obtained all data from public websites, the veracity of which is unknown. Some data were not available for all contestants, and it is likely this is differential (i.e., more successful candidates will have more written about them online than less successful ones as demonstrated by missingness for star sign for example). The data are limited to contestants on *Love Island*, UK; and therefore we are unsure of the generalizability of the findings to other country versions of the show (e.g., *Love Island Australia*).

Despite using data from all islanders, the sample size is nevertheless small and we are underpowered to detect predictors with small effect sizes. As a result of the sample size, overfitting likely occurred (the number of events per predictor was much less than required by rules of thumb) and we also importantly did not have a validation dataset to assess predictive performance of the score on [7]. Additionally, due to the small sample size, an out-of-sample cross-validation approach to assess the predictive accuracy would not return very meaningful results. Related to this we studied a number of potential associations, and used a nonconservative threshold to determine statistical significance. It is therefore likely that some significant associations resulted from the simple play of chance. The findings, therefore, need to be validated in another dataset.

There is a potential bias for two of the predictors that are based on events on the show (number of couplings and doing bits), in that these are more likely to happen, the longer an islander is on a show and the very nature of being on the show longer means that the islander is likely to be popular having been kept in by the public and/or fellow contestants. This potential bias is not obvious as, for example, there is not a linear relationship between number of couplings and show success (e.g., four and five couplings increased likelihood of success whereas three and six couplings did not) and it is also hard to disentangle given the nature and size of the dataset.

Our results should thus be regarded as speculative rather than definitive: they represent results from what can be done using online (‘big’) data. Further work is needed to validate the findings. We would explicitly call this to the attention of anyone thinking about adopting strategies such as doing bits to succeed as this is a decision that should not be taken lightly [8], and certainly not on the basis of a speculative chance of winning a TV show.

### Comparison with other studies

To the best of our knowledge, this is the only published study investigating factors predicting success on *Love Island*. Previous publications have discussed whether smoking behavior [9] displayed on screen may promote these behaviors in viewers. Reassuringly smoking did not appear to be predictive of show success.

## Conclusion & future perspective

We propose a novel score, DO-BITS, that provides an easy, practical tool to assess the individual likelihood of success on *Love Island*. After validation, the use of this simple score may potentially support decision making regarding whether or not to participate on the show and/or strategies to succeed if already participating on the show.

**Summary points**

- *Love Island* is a reality television program that has attracted a lot of attention, including participation from medical and scientific professionals.
- In order to help people decide whether to potentially make a life changing move to participate on the show, we sought to identify predictors of show success.
- A score predicting show success termed DO-BITS (Different coupling approaches [islanders pursuing one or many people on the show], Original islander [being on the show from the start], [being] Brunette, [having] Intimate relationships on screen, Tradesman [occupation before being on the show] and Short name [having a four-letter first name]) was developed.
- This simple, novel score provides a practical tool to assess the likelihood of success on *Love Island*.
